# More than meets the eye: The role of microglia in healthy and diseased retina

**DOI:** 10.3389/fimmu.2022.1006897

**Published:** 2022-11-29

**Authors:** Elisa Murenu, Maximilian-Joachim Gerhardt, Martin Biel, Stylianos Michalakis

**Affiliations:** ^1^ Department of Ophthalmology, Klinikum der Ludwig-Maximilians-Universität München, Munich, Germany; ^2^ Department of Pharmacy, Ludwig-Maximilians-Universität München, Munich, Germany

**Keywords:** microglia, retina, immune system, homeostasis, disease

## Abstract

Microglia are the main resident immune cells of the nervous system and as such they are involved in multiple roles ranging from tissue homeostasis to response to insults and circuit refinement. While most knowledge about microglia comes from brain studies, some mechanisms have been confirmed for microglia cells in the retina, the light-sensing compartment of the eye responsible for initial processing of visual information. However, several key pieces of this puzzle are still unaccounted for, as the characterization of retinal microglia has long been hindered by the reduced population size within the retina as well as the previous lack of technologies enabling single-cell analyses. Accumulating evidence indicates that the same cell type may harbor a high degree of transcriptional, morphological and functional differences depending on its location within the central nervous system. Thus, studying the roles and signatures adopted specifically by microglia in the retina has become increasingly important. Here, we review the current understanding of retinal microglia cells in physiology and in disease, with particular emphasis on newly discovered mechanisms and future research directions.

## Introduction

Microglia cells are the main resident immune component of the central nervous system (CNS), as opposed to those elements of the immune system that are circulating or do not reside in the parenchymal part of a tissue. First found by Franz Nissl at the end of the 19^th^ century and addressed as “rod cells”, it is only after the characterization of neurons and astrocytes that Ramon y Cajal’s student del Rio-Hortega used the term microglia to indicate a “third component” of the brain that was distinct from oligodendrocytes (1). The initial source of this cell population has found little consensus for many years, during which several researchers supported the theory of the neuroglial origin advanced by Ford Robertson and Ramon y Cajal (2–5), as opposed to del Rio-Hortega’s theory of the myeloid origin. The latter has been long controversial, only to be accredited when the expression of markers typical of macrophages, which originate from myeloid precursors, was confirmed also in microglia (6–11). Not surprisingly, microglia are considered the main resident macrophage population of the CNS (12).

The retina is the neural region of the eye where visual stimuli are detected and receive a first elaboration prior to being sent to the visual cortex. During late embryonic development, it consists of retinal progenitor cells organized in a neuroblast layer (NbL), which progressively give rise to all cell populations in a timely regulated manner (13, 14). At later postnatal and adult stages, this highly specialized tissue is organized in a well-characterized circuitry, consisting of nuclear and plexiform layers. Rod and cone photoreceptors, bipolar and ganglion cells belong to the first category and constitute outer nuclear (ONL), inner nuclear (INL) and ganglion cell layer (GCL), respectively (14, 15).

Other cell types residing in these layers are Müller glia (in the INL), horizontal (INL) and amacrine cells (within INL and GCL). A synaptic layer between ONL and INL as well as between INL and GCL constitute the outer (OPL) and inner plexiform layers (IPL), respectively (14, 15).

“The only thing I know is that I know nothing” - the famous Socratic paradox holds partly true for retinal microglia, on which relatively little is known compared to the brain counterpart. Hence, it comes to no surprise that most literature on retinal microglia simply relies on the assumption that this population performs the same tasks observed in brain microglia. However, already within the brain, at least part of the microglial population harbors diverse degrees of heterogeneity depending on their localization (16–19). Further complicating the scenario, microglia display a high context-dependent plasticity, making the classification of all acquired identities (according to morphology, molecular signature and function) a matter of active debate (20). As such, in this review we will step back to have an unbiased look specifically at the microglia population in the Mammalian retina and to address its known roles in retinal physiology and pathology. We will try to shed some light on recently discovered mechanisms and evidence from experiments carried out specifically in retinal microglia, all the while bringing the attention to the many persisting gaps of knowledge.

## Origin and consolidation of retinal microglia

### Origin and retina colonization

In comparison to the brain, pinpointing the original source and temporal appearance of microglia cells in the retina has proven more challenging, possibly for the physical separation of this compartment from other CNS areas and for the relatively reduced size of this population (21, 22).

Although the origin of retinal microglia has not been confirmed specifically, fate-mapping studies performed on murine models have shown that microglia population as a whole is generated by erythro-myeloid progenitors located in the yolk sac (23–30), in particular from a sub-population that does not express the transcription factor Myb (31, 32). Several studies have determined that a cohort of transcriptional regulators, such as Spi1 (encoding for the protein PU.1), Irf8 (10, 24) and Csfr1 (33–35) is crucial for microglia generation. The mutation or deletion of either of these genes impairs, and in some cases completely abolishes, the production of microglia.

**Figure 1:**
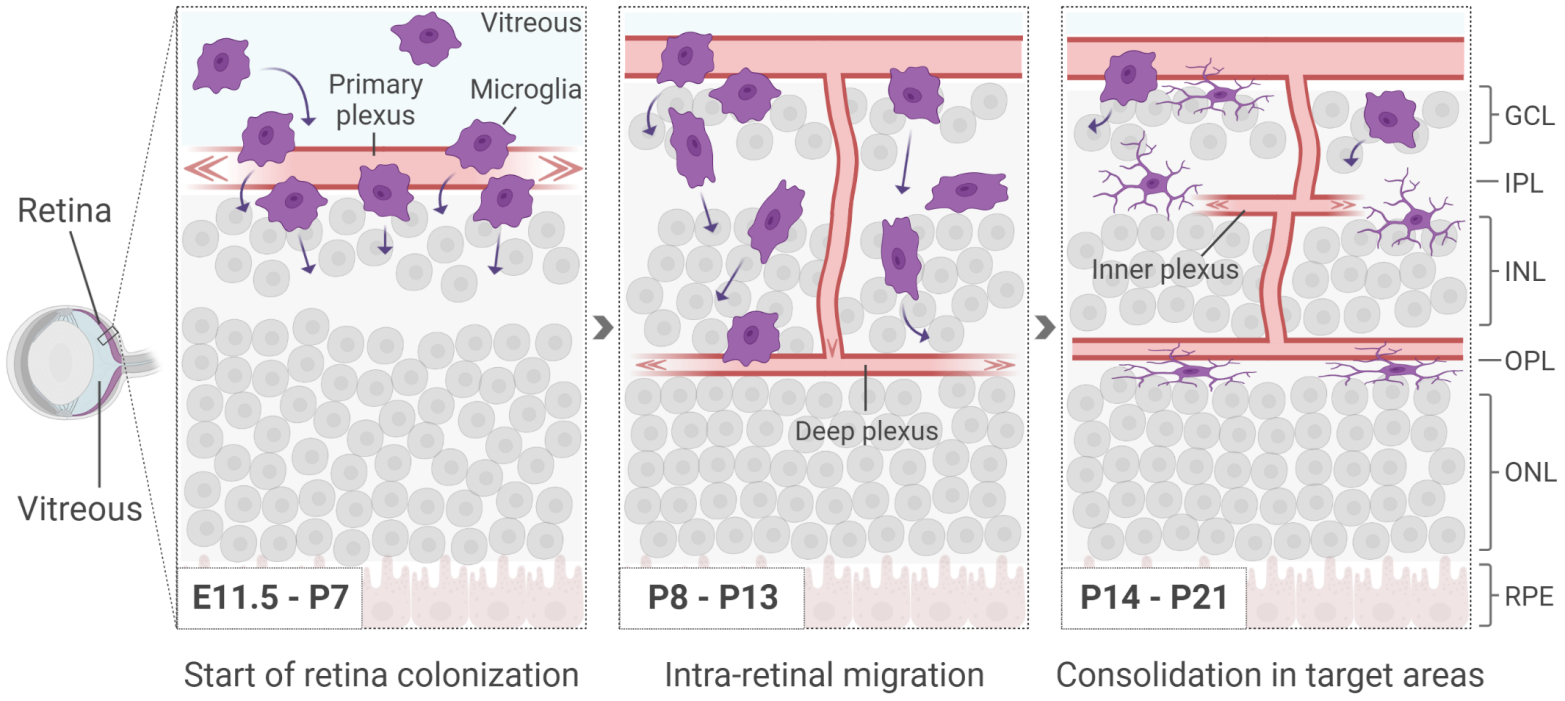
Microglia start appearing in the retina around E11.5, before the formation of the inner vasculature. At early postnatal days, as the deeper vasculature plexus develop, microglia infiltrate the retina in a radial manner, colonizing first the IPL and subsequently the OPL. Retinal cells are shown simplified. GCL, ganglion cell layer; IPL, inner plexiform layer; INL, inner nuclear layer; OPL, outer plexiform layer; ONL, outer nuclear layer; RPE, retinal pigmented epithelium.

After differentiation, microglia invade the CNS in two consecutive waves. Following the first wave, microglia cells reach the brain around embryonic day (E)8.5. The colonization of the retina, instead, has been determined as early as E11.5 in rodents (10 weeks of gestation in humans), though earlier time-points have not been extensively addressed (36–39) (**Figure 1**). At this time, vascularization has yet to take place and the vitreo-retinal interface and/or the ciliary margin zone (CMZ) have been indicated as main entry routes (36, 40, 41). The second wave is observed around birth, when the retinal vasculature is in the process of being established (**Figure 1**). Initially, microglia cells migrate tangentially at the interface with the ganglion cell layer, where the primary plexus of the vasculature is located (7, 42). Until postnatal day (P)10, they progressively invade the retinal tissue migrating in a radial fashion, in order to colonize the IPL first and the OPL later, while following the formation of the deeper vasculature plexus (7, 36, 40, 43, 44) (**Figure 1**).

The key drivers of the radial migration are not fully uncovered yet. However, microglia have been shown to be naturally attracted to sites with increased stiffness such as the ONL, a process referred to as durotaxis (45–47). Once the IPL and OPL have been colonized, microglia persist in these territories until adulthood, forming a network with a constant intercellular distance that is likely to be acquired *via* repulsive mechanisms (48). Additionally, microglia are found in the retinal nerve fiber layer (NFL) adjacent to ganglion cells and occasionally with their soma in the INL. The ONL is the only retinal region where no microglia are observed in physiological conditions (36).

### Repopulation routes and microglia turnover

The events underlying retina colonization have been additionally explored at adult stages, in order to shed light on the migratory behavior of microglia in a context where circuitries are already established and consolidated. To this end, murine experiments of pharmacologic or genetic ablation of microglia have shown that this cell population is capable of rapidly repopulating the retina and that the employed routes partly differ from those followed by microglia during development (49, 50). Indeed, following ablation at adult stages, most microglia cells origin from infiltration through the optic nerve and subsequent center-to-periphery migration, while repopulation occurs only to a lesser extent *via* invasion from the CMZ (49, 50). Cells that have already migrated, as well as cells that have survived ablation, further contribute to repopulation through local proliferation. Interestingly, migration within the retina seems to follow the same steps seen during development, with a radial distribution pattern starting at the interface of the GCL followed by colonization of IPL and OPL (49, 50). Thus, while cellular tropism *per se* relies on rather conserved mechanisms, and the retina environment remains seemingly permissive to (re)colonization even at adult stages, the cells contributing to repopulation are different in identity and location source, and mainly consist in microglia cells within the optic nerve that have survived the ablation as well as, to some extent, infiltrating macrophages (49, 51–53).

Supporting this evidence, retinal microglia show very little turnover in physiological conditions (29, 30, 54, 55), indicating that local proliferation might be mainly restricted to extreme cases of retinal tissue alteration and/or microglia depletion. Indeed, past studies making use of irradiation as a mean to deplete microglial population suggested a higher turnover rate promoted by infiltration of peripheral monocytes (56, 57). However, these results were the direct consequence of the impairment of the blood-retinal barrier caused by the experimental methods adopted, rather than of physiological turnover.

Thus, retinal microglia appear as a highly stable cell population, which, however, retains the ability to properly colonize and integrate in the adult retina following an alteration of its numbers.

### Retinal microglia morphotypes across development

**Figure 2:**
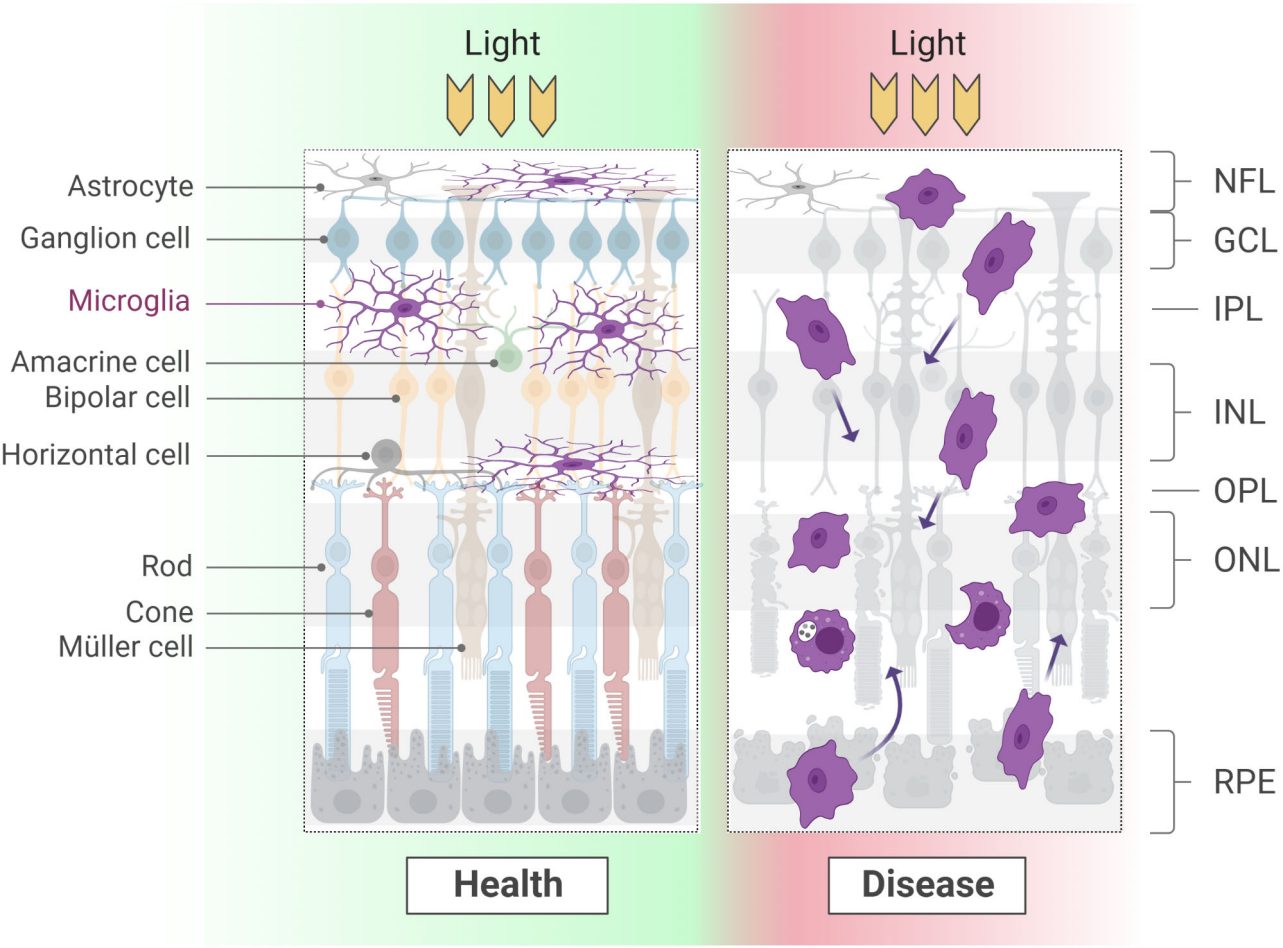
Microglial morphology varies considerably according to the developmental and physio-pathological state of the tissue. At adult stages, a long and thin arborization confers microglia the ability to scan large areas of the surrounding environment. When a degenerative process starts, microglia retract the branches and display high motility and phagocyting activity. Of note, while in health microglia occupy the synaptic layers and line ganglion cells fibers, during degeneration these boundaries are lost. NFL, nerve fiber layer; GCL, ganglion cell layer; IPL: inner plexiform layer; INL, inner nuclear layer; OPL, outer plexiform layer; ONL, outer nuclear layer; RPE, retinal pigment epithelium.

During the processes leading to retina colonization and proper integration, as seen across development, microglia cells adopt different morphologies. Similarly, the pathophysiological condition of a tissue and the location within the CNS correlate with microglial differences in size, polarization and complexity of arborization. In the retina, the first microglia cells entering the tissue are amoeboid in shape (**Figure 1**). Once at their final destination, their morphology changes either to a shape with long radial ramifications (within the IPL) or an elongated shape with highly polarized ramifications (within the OPL; **Figure 2**) (22, 47). The ramified morphology established during development has been indicated as a sign of quiescence, leading to the definition of “resting microglia”. Though the term might suggest the contrary, microglia in a resting state are rather active in tissue surveillance, constantly scanning the environment for any sign of physiological or pathological alterations (58–64). These processes are thought to be mediated by several extracellular cues, as already extensively described in other literature reviews (65–67). Examples of such cues are Cx3CL1, CD200 and ATP/ADP, which are detected by microglial receptors Cx3CR1, CD200R and P2RY12, respectively (68–73). Although it was suggested that the differential tissue stiffness of INL and ONL may impose the degree of microglial polarization (47), it remains unclear whether in homeostasis microglial morphotypes merely reflect the diversity a) in the tissue environment, b) in the functional states in response to local cues (59, 74–79), c) in the intrinsic molecular program, or d) in a mix of all of these. The evidence of distinct molecular signatures for some microglia sub-populations in various areas of the CNS might support the presence of a diverse molecular program defining not only retinal microglia as a whole (16–19, 30, 79–81), but also microglia within the same tissue, i.e., in IPL versus OPL regions. Recent findings have revealed that only IPL microglia cells depend on the Csfr1-IL-34 signaling (29), further prompting the importance of non-cell autonomous cues in the definition of microglial states and, possibly, identities. Additional studies are necessary to fully uncover the molecular program characterizing the IPL and OPL sub-populations, the factors influencing the correct positioning in either one of these plexiform layers and the distinct roles they might play in health and disease.

At postnatal and adult stages, the acquisition of an amoeboid morphology is instead associated with a reactive state, following the onset of a degenerative pathology or the occurrence of an injury (82–85) (**Figure 2**; see section Roles of microglia in pathology).

## Roles of microglia in physiology

As discussed in the previous section, the presence of microglia in the retina has been determined as early as E11.5. From this point, the density of microglia in the mouse retina increases to such an extent that by P7 it accounts for double the density observed in the adult (22, 36, 86). As local cell proliferation has been ruled out during this phase, the likely explanation for the observed increase is the ingress of new microglia cells (36). Conversely, a decrease in density follows during the second postnatal week and it has been accredited to the expansion of the retinal size together with the absence of new microglia cells entering the tissue (36, 41). Of note, changes in absolute numbers are not known and selective cell death at these stages has not been ruled out. During physiological aging, instead, the number of microglia cells increases in the subretinal and perivascular space (87).

In order to elucidate the roles retinal microglia play during development, the attention has been turned to the physiological events taking place in the retina during microglia invasion and during their changes in density, using the acquired knowledge on brain microglia as reference. In the following sections, we will mainly address the result of studies in the murine retina, unless differently specified.

### Vasculature development

As addressed in section Origin and retina colonization, early microglia progenitors colonize the retina in two consecutive waves. Particularly the second wave takes place around the time retinal vasculature appears and shares with it the same pattern of invasion, i.e., from vitreo-retinal interface to deep retina layers (7, 36, 40, 43, 44, 88). This temporal and spatial correlation raised the question of a potential link between microglia cells and formation of new blood vessels in the retina. Indeed, during development microglia are found in close proximity to nascent blood vessels and extend branches to contact endothelial cells and pericytes (37, 88, 89). Several receptors, including Mas1 and Notch1, are likely to mediate the active localization of microglia in these regions, as mice lacking their expression show reduced numbers of microglia near sprouting blood vessels and general defects in vasculature formation (90, 91).

Further substantiating a role for microglia cells in retinal vascularization, inducing microglia ablation around birth causes a reduction of the area covered by blood vessels and a general impairment of the vasculature, whose growth is, however, rescued when new microglia cells are transplanted (88, 92). Transforming growth factor (TGF) β1, in particular, has been indicated as the main microglial mediator of vascular growth and its tight regulation is key in promoting physiological vascularization (47, 93).

During postnatal development, microglia are additionally involved in vasculature regression to help refine the blood vessel landscape, as seen both in mouse (94) and human retina (92). This process seems to be mediated by several signalling factors, such as Wnt, macrophage colony stimulating factor (M-CSF) and angiopoietin (94–97). In addition to vascular regression, microglia/macrophages mediate suppression of blood vessel branching *via* vascular endothelial growth factor (VEGF)-Flt1 axis, acting in conjunction with non-canonical Wnt signalling (98).

All in all, experimental proof supports the contribution of microglia in the development of retinal vasculature, especially by exercising an important inhibitory role on vascular sprouting. Of note, astrocyte progenitors are, instead, known to actively promote vascularization (40, 99, 100). Therefore, microglia are seemingly involved in vasculature development also indirectly, *via* regulation of the numbers of retinal astrocytes (101).

### Synaptic refinement

Aside from the already mentioned role during vascularization (47, 88, 92, 94–96, 98, 102–104)(see previous section), shaping the nascent synapses is considered one of the main functions of microglia. Synapses in the retina are established on the one hand among photoreceptors, horizontal and bipolar cells, on the other hand among bipolar, amacrine and ganglion cells. At late embryonic stages in the murine retina, the synaptic marker synaptophysin is detected in the photoreceptor outer segments (13). As early as P5, the same marker can be found in both IPL and cones, becoming restricted to pre-synaptic terminals of photoreceptors from P10 and, finally, being detected only in IPL and OPL from the second postnatal week (13, 105). Arborizations and synapses keep on being consolidated and refined from eye opening (P13-14), following an activity-dependent scheme and resulting in more spontaneous synaptic inputs (106).

Temporally and spatially, microglia invasion of the retina follows the same pattern seen in the development of cell-cell connections. Together with this observation, the known role of brain microglia in pruning of synapses during development (106–108) prompted the idea that similar mechanisms might concur to shape the retina circuitry. In this regard, it was shown that microglia regulate the extent to which the dendrites of horizontal cells are extended *via* the complement protein C1q (107). As such, C1q-depleted mice show a progressive invasion of horizontal cells dendrites into the ONL from P13 on, at the time when the synapses in the OPL are being consolidated. While any other direct evidence of retinal microglia involvement in pruning during development has yet to be uncovered, brain microglia have been described to refine presynaptic terminals of ganglion cells in the lateral geniculate nucleus (LGN) through complement protein activity (108, 109). Interestingly, the impairment of Cx3CL1-Cx3CR1 signaling in mice, particularly important for microglia activity (110), was associated with the extension of microglia branches inside the ONL and morphological and functional alterations in photoreceptors during early postnatal development, particularly in cones (111). Similarly, specific depletion of microglia at adult stages was shown to affect photoreceptor synapses and visual performance (112, 113). Of note, an additional role for microglia in axon guidance of ganglion cells through nerve growth factor (NGF) signaling was suggested (114), thus corroborating a multi-faceted spectrum of activities for microglia also in the context of the retina.

### Developmental cell death

Strictly interconnected with synaptic refinement, and, therefore, proper neuronal integration, is the regulation of cell numbers. The early developmental phases of the CNS have been associated with extensive cell death. Both cell autonomous and non-cell autonomous processes concur to eliminate exuberating cells and/or newborn neurons that fail to establish a proper connection with their targets (115, 116). Such mechanism also takes place within the retina, where three waves of programmed cell death (PCD) have been described: morphogenic, early neural and neurotrophic cell death (117–120).

Morphogenic cell death is found in the neuroepithelium of the eye primordia during embryonic development, peaking at E10.5 and extending until E14.5 (117, 121). Its name reflects the contribution of this PCD to the processes that shape the optic cup during this time. Early neural cell death, instead, occurs during E15-E17 and involves retinal ganglion and amacrine cells, namely the first cell types derived from retinal progenitors (122–124). NGF signaling acting through p75 receptor has been indicated as the trigger of this apoptotic wave (125). Finally, late or neurotrophic cell death coincides with the establishment of connections with extra-retinal structures at postnatal stages and affects several cell populations (118, 126, 127). Between P4 and P10, TGF signaling triggers the correct expression levels of NGF, which, contrary to the detrimental effects mediated by p75 during embryonic development, promotes survival of retinal neurons, possibly through activation of TrkA receptor (128). The same is observed in the adult retina, where TGF-β1 is involved in the maintenance of ganglion cells (129). Of note, a bystander cell death effect has also been described at early postnatal stages (130).

The role of microglia throughout these steps is still mostly circumstantial. Microglia in early phases of retina colonization has been observed in proximity of dying cells (7, 39, 114). From E12.5 until birth, retinal microglia cells predominantly localize in the compartment containing already differentiated cells, while in the NbL almost 50% of microglia cells establishes contacts with newborn migrating ganglion cells. Interestingly, mainly non-apoptotic ganglion cells undergo phagocytosis after being marked with complement proteins (41), a process defined phagoptosis (131). However, a previous study indicated that microglia do uptake remnants of apoptotic ganglion cells that had been retrogradely labelled with Fluorogold or (4-[4-didecylaminostyryl]-N-methylpyridinium iodide (4Di-10ASP) (44). Thus, it is unclear whether microglia merely adopt a scavenging role or rather an active role in the phagocytosis of dying retinal cells.

Moreover, aside from regulating the number of ganglion cells, microglia seem to influence the number of other retinal populations, such as astrocytes (101).

### Cell survival and proliferation

In addition to cell death, proliferation and cell survival act as direct regulators of cell numbers. Similar to the previously addressed roles, recent evidence links brain microglia population and progenitor cell behavior. While a bidirectional cross-talk with neural stem cells and progenitors has been extensively described both during development and at adult stages (132, 133), the contribution of microglia to similar mechanisms in the retina is limited to selected examples. During early postnatal development, the number of proliferating progenitors can be increased by lipopolysaccharide (LPS) administration, whose primary effect is microglia activation and proliferation (86). Accordingly, microglia depletion results in decreased progenitor proliferation. Knockout of microglial-expressed gene *progranulin* (*grn*) in mice has been shown to result in a decrease in numbers of both microglia and retinal progenitors (86). In agreement with this, activated microglia have also been shown to promote cell survival in retinal explants from P10 postnatal mice, as interfering with their activation or promoting their ablation leads to decreased viability of retinal cells (134).

As already addressed, it is worth noting that microglia have also been associated with proper cone maturation *via* Cx3CL1-Cx3CR1 signaling and maintenance of cone homeostasis at adult stages (111, 112). Though intriguing, the nature of this selective relationship between microglia and cone cells has yet to be fully characterized.

## Roles of microglia in pathology

As befitting cells participating in the immune response of an organism, microglia adopt a more active (and overall, better characterized) role in pathology. Consequently, understanding the behavior of microglia in disease can help uncover additional functions adopted in physiological conditions.

Several factors expressed in the healthy mouse, rat and human retina serve as signals for microglia to remain in a non-reactive state, e.g., CD200 and Cx3CL1 (60, 70, 71, 135). The loss of retinal homeostasis resulting from a physical insult or a degenerative process causes an alteration in the levels of these and additional factors, thus leading to microglia activation and recruitment. These events correlate with the acquisition of an amoeboid morphology and a highly migratory behavior that brings microglia cells to invade nuclear layers (particularly the ONL) commonly devoid of immune cells (82–85). It was proposed that activated microglia adopt distinct transcriptional features depending on whether a mostly pro-inflammatory (M1) or anti-inflammatory (M2) program is initiated. However, these definitions are largely stereotyped and possibly more suitable for monocytes (20, 74, 136–140). The rapid expansion of high-throughput methods, particularly single-cell RNA sequencing, has recently enabled a more granular characterization of several phenotypes (and morphotypes) acquired by microglia in different brain areas and conditions (18–20, 138, 141, 142). Most of these definitions, including disease-associated microglia (DAM), proliferative-region-associated microglia (PAM), axon tract-associated microglia (ATM) and cd11c+ microglia, have recently found correlation also in mouse retina, specifically at postnatal stages during developmental apoptosis and in several paradigms of degeneration (143–149).

Although categorizations often suggest stereotyped responses in well-defined contexts, the role of retinal microglia during degeneration is multifaceted. Here, we will review the current literature on microglia in the context of severe sight-threatening eye diseases that are predominantly associated with retinal degeneration.

### Retinitis pigmentosa

Among inherited retinal diseases (IRDs), retinitis pigmentosa (RP) is the most prevalent form and includes a spectrum of diseases that can be generally categorized in syndromic (= affecting various organs) and non-syndromic (affecting only the retina) (150, 151). Initially characterized by rod photoreceptor loss in a peripheral-to-central gradient, the degeneration soon extends also to cones. RP patients notice nyctalopia and impaired dark adaptation already at early stages of the disease, and suffer from a progressive, usually concentric, visual field impairment. Currently, no remedy exists for the various forms of RP, although several different approaches have been tried and many others are currently under investigation (152, 153). The sole important exception is represented by voretigene neparvovec, an AAV-based gene therapy specifically designed for the treatment of retinal dystrophies caused by *RPE65* bi-allelic mutations, although the latter ones account only for a very small proportion of all RP cases (152–154).

Early RP is characterized by progressive loss of rod photoreceptors. As discussed in the opening of this chapter, the loss of photoreceptors *per se* deprives microglia of signals important for their homeostasis, though sudden hyperoxidative environment, oxidative stress and alteration of retinal metabolism may also concur to foster and sustain microglial response (155–160). Similar to what happens throughout the CNS, retinal microglia usually respond by adopting an amoeboid morphology and migrating to the area of insult, ultimately proliferating to amplify their numbers and acting to resolve the ongoing degeneration. In the context of RP, as seen in mouse models, activation of microglia cells leads to their migration into the ONL, production of pro-inflammatory cytokines and phagocytosis of viable photoreceptors, which further contributes to disease progression (65, 161–164).

Thus, it is reasonable to speculate that activating pathways involved in microglia homeostasis might concur to ameliorate the effects of the degeneration. In this scenario, Cx3CL1 administration to well-established mouse models of RP (*Pde6b^rd1^* and *Pde6b^rd10^* mutants, also known as rd1 and rd10, respectively (165, 166), has been shown to improve survival of photoreceptors, though the positive effects may not be necessarily mediated by microglia (167, 168). In agreement with the homeostatic role of Cx3CL1-Cx3CR1 signaling, loss of photoreceptors is exacerbated in mice lacking Cx3CR1, possibly due to the inability to activate downstream pathways that would normally maintain microglia in a resting state both in mice and humans (72, 162). A similar result was described for insulin growth factor (IGF-1), whose anti-apoptotic effect seems to be mediated by newly recruited microglia cells (169). In addition to Cx3CL1 and IGF-1, TGF-β1 administration has also been shown to ameliorate cone survival in mice by reducing the pro-inflammatory effect of microglia (170).

Although involved in removal of hindered photoreceptors and in scavenging of cellular debris, microglia have been shown to additionally phagocytize seemingly healthy photoreceptors (171–173), as already observed for ganglion cells during development (41). These observations prompted to think that microglia depletion, rather than mere modulation, would considerably hinder disease progression. Indeed, interfering with phagocytosis or selectively inducing microglia cell death has been shown to ameliorate retinal cytoarchitecture and overall visual function in *Pde6b^rd10^* mice (171). However, a recent work has challenged this view by indicating a protective effect of microglia on cones in the same mouse model (174), as suggested by the discussed role of microglia in cone homeostasis (111). In rats, treatments affecting microglia numbers have resulted in reduced visual function, as a consequence of an increased apoptotic rate in retinal neurons (172, 175). Partially explaining this protective role, microglia are not only involved in phagocytosis of stressed photoreceptors, but also in refinement and clearance of improper synaptic connections attempted by bipolar cells with remaining photoreceptors. Such role seems to be mediated by C1q expression in the dendrites of bipolar cells (172), while both complement protein C3 and its receptor (C3R) have been implicated in pruning of photoreceptor synapses (173). Genetic depletion of either C3 or C3R aggravated photoreceptor death in the already affected retina. Similar results hinting at a neuroprotective role of microglia were obtained in a mouse model of retinal detachment (176) and of excitotoxicity (177). Interestingly, signs of excitotoxicity were also found in *Pde6b^rd1^* mutants, possibly justifying similar microglia responses in different degenerative contexts (158, 178).

Thus, it is conceivable that microglia attempt to contain the degeneration by targeting both dying and viable (albeit already compromised) photoreceptors. Impairing microglia numbers or their phagocytic ability soon after infiltrating the ONL leads to an accumulation of stressed and/or dying photoreceptors, which seems to transiently ameliorate the progression of the disease. In later stages of the disease, however, the ONL is affected in the same way as in untreated retinae, possibly due to the inability of the remaining photoreceptors to maintain proper connections with bipolar cells. Microglia cells surviving the depletion might also concur to aggravate the degeneration when their phagocytic ability is impaired.

### Glaucoma

Aside from IRDs, the most common cause of irreversible blindness as of 2020 is glaucoma (179), which encompasses a spectrum of heterogenous diseases whose underlying etiology is still largely unknown (180, 181). The common leitmotif in affected patients is a gradual degeneration of the retinal ganglion cells, the associated optic nerve and the supporting connective tissue, which become ophthalmologically apparent as an excavation of the optic disc (180, 181). An elevated intraocular pressure (IOP) is the most common risk factor and, currently, the main target of treatment (182).

Although the principal factors driving glaucoma are still under investigation, the involvement of the immune system in the pathology is rather established (183–191). Indeed, complement and endothelin systems have been found to be overrepresented in both patients and animal models of glaucoma even before the appearance of other phenotypic effects (183, 190, 192–210). Interestingly, antagonizing C1qa protein produced by ganglion cells, microglia and macrophages exerts positive effects on glaucoma progression (183, 195, 197, 211), whereas mice genetically depleted of C3, produced by astrocytes, show an aggravation of the phenotype (196). In contrast, attenuation of C3 production specifically in the retina resulted in increased survival of ganglion cells, suggesting that the levels of certain complement factors in this locally restricted context play a complex role in the onset and progression of the pathology (194).

In the context of glaucomatous optic atrophy, microglia localize to the optic nerve head, where they locally proliferate and release inflammatory molecules such as tumor necrosis factor (TNF)-α and TGF-β (212–214). Their recruitment precedes or coincides with the actual onset of degeneration, possibly as a consequence of the local complement accumulation (191, 208, 215). Following ganglion cell damage and subsequent degeneration, retrograde labeling of the optic nerve leads to simultaneous labeling of retinal microglia, indicating a central role of the latter population in the clearance of debris and removal of dying ganglion cells (216, 217). Interestingly, in models of unilateral glaucoma induction, e.g., *via* laser-induced ocular hypertension (OHT), microglia cells have been shown to react in the unaffected contralateral control eye, though to a lesser extent (218–221). In addition to the canonical signs of activation, such as increase in overall cell size, migratory behavior and reduction in arborization, the extension of microglial processes from the OPL to the photoreceptor outer segments is particularly pronounced in the control eye (219, 220). Such feature has also been observed in some mouse models of age-related macular degeneration (AMD) (222). These activated microglia cells seem to adopt a more pro-inflammatory state, as the expression of M2-polarization marker Ym1 was not detected (221, 223). However, microglia have been shown to assume a rather anti-inflammatory identity in other glaucoma models (224). These observations highlight the importance of both context and choice of disease models and further substantiate the complexity and plasticity of the microglia population in response to different stimuli.

Not less importantly, microglia have been shown to facilitate monocytes recruitment and infiltration, two processes that are usually mediated by the presence of the chemokine Ccl2 in the tissue and the expression of its receptor Ccr2 on these cells (225), though also activated microglia is able to express Ccr2 in particular degenerative conditions (226). Monocytes have been shown to infiltrate the retina of rodent glaucoma models and to promote degeneration. Accordingly, their depletion *via* radiation treatment, or inhibition of their entry *via* chemical treatment or *Itgam* knockout (also known as Cd11b, the alpha subunit of C3R), results in extended neuroprotection (187, 188, 209, 227). Similar results were obtained by hindering monocyte recruitment through the inhibition of TNF-α or IL-1β activity (228). Of note, while low doses of Ccl2 promote survival of retinal ganglion cells, seemingly mediated by microglia, studies performed in rats suggest that high doses accelerate their loss (229). Thus, a key aspect of microglia behavior is the context-dependent modulation of its state/activation. However, the mechanisms linking Ccr2 to such dual role are not fully uncovered.

### Age-related macular degeneration

AMD represents the most common cause of irreversible central vision loss and its prevalence is expected to increase dramatically within the next decades (179, 230, 231). Early and intermediate AMD is characterized by the formation and expansion of extracellular deposits (so-called drusen) beneath the retinal pigment epithelium (RPE) or in the subretinal space (231, 232). In advanced stages, AMD either progresses to a neovascular (wet) form or leads to progressive cell death without any neovascular component (dry AMD with geographic atrophy). Wet AMD is associated with the development of new blood vessels (choroidal neovascularization, CNV), leading to rapid vision loss through exudative macular edema, subretinal/intraretinal bleedings and progressive scarring of the central retina. Dry AMD progresses to geographic atrophy, characterized by a complete loss of both photoreceptors and RPE in the macula (233). Importantly, these two late forms of AMD are not completely exclusive, as either one can eventually evolve into the other (231, 232).

Similar to glaucoma, the presence of complement proteins in drusen is *per se* a strong indication of the involvement of the innate immune system in AMD and, indeed, macrophages and microglia have been found in its association (234–239). This observation is especially true for the neovascular form of AMD, where complement has been shown to be associated with an increase in the levels of the pro-angiogenic VEGF (234, 240), partly contributed by microglia and macrophages (241–246). Anti-VEGF drugs are indeed to date the only approved therapy for AMD, though only the exudative form is treatable (247, 248).

Microglia exceptionally found in subretinal space is one of the hallmarks of AMD and is thought to have both protective and detrimental effects on RPE and photoreceptors (55, 249–253). The colonization of this area is achieved by the mobilization and subsequent proliferation of the retinal microglia population as well as infiltration of circulating monocytes (30, 55, 64, 243, 253–258). Aged mice lacking Ccr2, its ligand Ccl2 or Cx3CR1 have been shown to develop retinal lesions that are reminiscent of human AMD (240, 259–263) and, indeed, mutations in *CX3CR1* gene have been found also in patients affected by AMD (259, 264–267), but not mutations in *CCR2* (268). The infiltration of microglia in the subretinal space of these mouse models is likely to induce para-inflammation and determine cellular death (260). Moreover, microglia could also be involved in (and/or be activated by) the production of reactive oxygen species (269), which, together with changes in cellular metabolism, are considered among the possible causes of AMD (270–272). Notably, another cohort of studies showed that mice deficient for Cx3CR1 and/or Ccl2 do not develop AMD and that only the latter mouse line displays microglia/macrophages accumulation in the subretinal space (273). Such an accumulation of lipid-bloated immune cells can be easily mistaken for AMD-related lesions during ophthalmologic inspections, but would rather be a natural consequence of aging, as observed in aged control mice (87, 261, 262, 274). Possibly unifying these seemingly contradictory results is the discovery of the confounding mutation rd8 in C57Bl6/N mice, which were used to generate most of these mutant mouse lines (275). However, a common view on the matter has not been achieved and the subject remains under debate (222, 276, 277).

The presence of both microglia and infiltrating monocytes/macrophages, which share many molecular characteristics and functional properties, renders distinguishing their individual contribution to diseases particularly challenging (278). Nevertheless, potential treatments alleviating or hindering the progression of AMD through the modulation of the immune system are likely to act on both cellular populations. Among those, interferon-beta (IFN-β) administration in the laser-induced model of CNV was shown to attenuate the phagocytic activity of both microglia and macrophages, whereas the knockout of IFN-β receptor (*Ifnar1*) promoted neovascularization (279). Similarly, the reduction or loss in TGF-β signaling has been associated with microglia reactivity and aggravation (but not causation) of AMD (254). However, the literature on this topic is still largely controversial, partly due to the high degree of pleiotropism displayed by TGF-β signaling both in animal models and in patients (280–286).

Furthermore, the blockade of adenosine receptor A_2A_R activity has been shown to decrease reactivity and complement deposition in human microglia cells, all the while enhancing clearance of photoreceptor debris and improving cell survival (287).

Interestingly, the endolysosomal system can also be manipulated in order to alleviate wet AMD conditions. Indeed, our group has recently shown that interfering with two-pore channel 2 (TPC2) function on endolysosomes in a model of laser-induced AMD leads to reduced recruitment of retinal microglia/macrophages (288). Moreover, *Tpc2* knockout determines a reduction in neovascularization *via* a decrease in both VEGF and IL-1β levels, the latter being a pro-inflammatory interleukin that has been already associated with photoreceptor degeneration and pathological neovascularization (288–290). Further studies are currently undergoing to better characterize the mechanisms linking TPC2 to the beneficial effects observed.

### Retinal vascular disorders

Microglia are involved in multiple additional retinopathies, although their role is not always fully characterized. Among those whose incidence is expected to increase in the coming years there is diabetic retinopathy (DR) – a condition triggered by systemic diabetes and initially determining vascular abnormalities in the retina (291). Both in mice and human, ganglion cells, amacrine cells and, to a lesser extent, photoreceptors undergo apoptosis already at early stages (292–294), but signs of neurodegeneration may occasionally appear even before changes in retinal vasculature become appreciable (295–297). At advanced stages, ischemia and reactive oxygen species aggravate the scenario by triggering *de novo* vascularization in vitreous and vitreo-retinal interface (298, 299). As a consequence of all these vascular alterations, vision loss ensues from diffuse edema, in particular in the macular region (298).

*Per se*, ischemia and hyperglycemia are potent activators of microglia, as indicated in several *in vitro* studies (300) and further observed in mice (88, 301–303) and rats in which type 1 diabetes was induced with streptozotocin (STZ) (89, 304–308) as well as in human patients (294, 309, 310). As described for other pathologies, activation includes events like proliferation, retraction of processes and acquisition of an amoeboid shape. In DR, microglia undergo these morphological changes before cell death is observed, supporting their involvement at very early stages, but not clarifying their role in disease onset and progression (311). Interestingly, in a mouse model of DR, systemic and local level of cytokines (such as IL-4, IL-13, TNF-α and IL-1β) indicated that microglia cells initially engage in an anti-inflammatory response, which is then almost completely lost in favor of a pro-inflammatory response at later stages (312). Thus, while microglia might not contribute substantially to DR onset, they rather sustain its progression, but only when attempts aiming at quenching the increasing inflammation are exhausted.

Retinal microglia have been shown to contact pericytes and blood vessels and regulate blood flow through Cx3CL1-Cx3CR1 axis, particularly by promoting vasoconstriction when the pathway is activated (37, 88, 89). At early stages in rat models of DR, in the absence of any sign of activation, microglia increase contact with capillaries and pericytes, thus causing an overall reduction in retinal blood flow (89). However, in a similar time window, a different work has determined that fractalkine levels in the retina are lower, with consequent increase in retinal inflammation (308). Further studies are required in order to assess the contribution of Cx3CL1-Cx3CR1 axis in DR and the associated role of microglia.

Similar to all other retinopathies, alterations in the complement system have been described for patients affected by DR and provide an additional link between microglia activity and disease progression (313, 314). Interestingly, though the mechanisms are not entirely elucidated, IgG-laden exosomes in the plasma have been found increased in DR and associated to complement activation (315). Additional efforts are needed to pinpoint the correlation between exosomes and DR onset.

Aside from DR, retinal vascular occlusion (RVO) is a common cause of vision loss, with vein occlusions being more common than arterial occlusions. The prevalence of both vein and arterial occlusions rises with age and is strongly associated with cardiovascular risk factors such as hypertension, diabetes and cardiovascular disease (316). Typically, an ischemic injury results from impaired blood circulation and leads to inflammatory responses, including microglia activation and proliferation. Moreover, cell death eventually affects retinal ganglion cells, which are particularly vulnerable to ischemic conditions (317–324).

In a well-established rat model of high intraocular pressure, a strong increase in retinal microglia was observed following an ischemic insult and suggested the involvement of this cell population in RVO (318, 322). As such, it was speculated that microglia depletion and/or modulation could result in reduction of the local inflammation and general neuroprotection. Indeed, microglia depletion in mice with experimental branch RVO exerts a protective effect on retinal ganglion cells (325–327). Additionally, modulating microglial activity *via* selective inhibition of A_2A_ receptor prevents microglia-mediated neuroinflammation and protects retinal ganglion cells from transient ischemic injury (322, 328). Similar neuroprotective effects are exerted by treatment with the antibiotic minocycline, specifically *via* induction of IL-4 expression and polarization of microglia/macrophages towards an M2 phenotype, as observed in mice with induced retinal ischemia (324).

Thus, these examples illustrate the relevance of microglia in disease development and its therapeutic potential in retinal vascular occlusion. Inhibition of retinal microglia may represent a promising approach for modulating inflammatory responses and/or promoting neuroprotection.

## Discussion

Microglia have been in the spotlight since their discovery, but, despite receiving considerable and increasing attention over the years, many aspects of their biology and behavior remain unknown. From a developmental standpoint, it is interesting to notice how the main resident immune cell population arises from a different embryonic source from all other neural cells. This aspect imposes microglial progenitors to migrate extensively in order to colonize their neural areas of destination, while neurons, astrocytes and oligodendrocytes are generated *in situ* and are subject to more local migratory events. Provided that retinal microglia belong to the same population colonizing the rest of the CNS, an assumption that has not been corroborated by evidence yet, this feature translates into a delayed invasion of the retinal anlage compared, e.g., to the brain. However, the process still happens in a timely manner and precisely when synaptic connections are being developed within the retina. The latter correlation has been often pointed out, and although to date it has not been sufficiently explored, it could help to answer some questions regarding the temporal and spatial cues underlying this migration.

A second aspect worth highlighting is the complementary role adopted by macroglial cells in immune reaction. Particularly in the brain, part of the tasks seen for microglia are, in fact, mediated by astrocytes, with which microglia establish a tight and continuous cross-talk (329, 330). *Per se*, the fact that astrocytes already actively participate in the immune response program prompts the question of the evolutionary decision to rely on the non-neuroectoderm derived-microglia to cover and/or initiate most immune-related functions in the brain. Specifically for the retina, this point concerns mostly Müller cells, since astrocytes are only found at the interface of ganglion cells and are, therefore, physically separated from most retinal cells (65, 331). However, Müller cells and (cerebral) astrocytes are two biologically distinct cell types that only partially share functions, in turn suggesting that microglia in the retina might act differently or require different partners to accomplish the same tasks as microglia in the brain. Given the dissimilar cellular composition of the retina compared to the brain, one example being the exclusive presence of Müller glia in the former and of oligodendrocytes in the latter, and the segregation of retinal microglia in specific compartments (IPL and OPL) in contrast to the rather homogeneous distribution in the brain, it is conceivable that a regional distinction persists and concurs to define the identity of microglia subpopulations. Additionally, retina and brain environments differ in other physical properties, such as extracellular matrix composition and vasculature coverage (332–335). Together with the cellular composition, which is reflected by different cellular players (e.g., Müller cells *vs*. oligodendrocytes) and overall proportion of cellular subtypes (e.g., neurons *vs*. glia), differences in these aspects confer unique stiffness landscapes as well as specific metabolic environments in brain and retina, which could influence both microglia regional identity and their behavior in disease (336–338). With this in mind, the implicit assumption that has driven and continues to drive the field, namely that the same cell type performs the same tasks regardless of the CNS region, albeit being sensible, must necessarily find more experimental support. As pointed out in this review, a first publication ended up underlying differences in IPL versus OPL microglia (29). Nevertheless, it is not clear which additional cue defines these two spatially segregated populations and guides their positioning during early development. Similarly, the contribution of microglia to physiological events in the retina, such as circuitry refinement and tissue homeostasis, has found little evidence to date.

The role of microglia in pathology has instead received more attention. Though the core of the microglial response is in principle quite stereotyped, i.e., by secreting and/or reacting to specific cytokines (or the lack thereof), exploiting the complement system, displaying hypertrophy and process retraction, adopting a highly migratory and phagocytic phenotype to remove affected cells, several differences persist depending on the pathology and the strategy used to model it. Of note, abolishing microglia or interfering with its function does not necessarily result in an improvement of the degeneration. An explanation for this seemingly counterintuitive effect could reside in the versatility of microglia, which can adopt a detrimental or a pro-survival role in a context-dependent manner. An additional explanation could be the beneficial functions that microglia have in homeostasis and that are lost following its depletion. Cone photoreceptors, in particular, seem to benefit from the presence of microglia, as demonstrated by studies underlying the involvement of the latter ones in their maturation and maintenance and the development of structural abnormalities when microglia are depleted. These findings suggest a very specific connection between these two cell types, although the nature and underlying mechanisms of this relationship have not been extensively addressed.

Given the high degree of complexity and variety in microglial behavior, leading to both beneficial and detrimental effects in the degenerative context, therapeutic strategies could benefit from an approach in which microglia are modulated/repurposed rather than depleted. Indeed, not only would depletion harbor the risk to aggravate and accelerate the ongoing degeneration, as observed in several models of retinal dystrophy, but it would also not be easily achieved. Recent studies focusing on the ablation of the retinal microglia population have acknowledged a rapid repopulation, triggered by infiltration and proliferation of microglia having survived the treatment. Since monocytes/macrophages have been shown to be able to infiltrate the retina and become resident microglia cells (55), strategies focusing on microglia depletion might bear limited effects in those contexts where a pro-inflammatory function of microglia is observed. Conversely, treatments that would end up redirecting to a more neuroprotective phenotype, though of difficult application, could result in increased neuronal protection and would not deprive surrounding cells of important homeostatic mechanisms attributed to microglia.

Further studies are required to shed more light on the functions of microglia in physiology, specifically in the retina, and on the singularities of retinal tissue compared to the rest of the CNS. While microglia populations across the CNS share many common features, different contexts may directly or indirectly impose distinct characteristics and novel functions. In parallel, precious information can be inferred from studying the immune reaction in retinal disease and from phenotyping microglial behavior in response to different pathologies.

All in all, many gaps await to be filled, making the field undoubtedly exciting, but all the while urging the community to turn a well-earned attention to retinal microglia.

## Author contributions

EM and SM contributed to the conception of the manuscript. EM wrote the manuscript. M-JG, MB and SM contributed to writing selected part of the manuscript. All authors contributed to manuscript revision, read, and approved the submitted version.

## Funding

This work was supported by Deutsche Forschungsgemeinschaft (MI 1238/4-1 and SFB1309).

## Acknowledgments

We thank Dr. Hanaa Ghanawi for insightful comments on the manuscript. Figures were created with BioRender.com.

## Conflict of interest

The authors declare that the research was conducted in the absence of any commercial or financial relationships that could be construed as a potential conflict of interest.

## Publisher’s note

All claims expressed in this article are solely those of the authors and do not necessarily represent those of their affiliated organizations, or those of the publisher, the editors and the reviewers. Any product that may be evaluated in this article, or claim that may be made by its manufacturer, is not guaranteed or endorsed by the publisher.
